# Climbing the mountain: experimental design for the efficient optimization of stem cell bioprocessing

**DOI:** 10.1186/s13036-017-0078-z

**Published:** 2017-12-04

**Authors:** Derek Toms, Rob Deardon, Mark Ungrin

**Affiliations:** 1 Department of Comparative Biology and Experimental Medicine, Faculty of Veterinary Medicine, University of Calgary, 3280 Hospital Drive NW, Calgary, T2N 4Z6 Canada; 2 Department of Production Animal Health, Faculty of Veterinary Medicine, University of Calgary, 3280 Hospital Drive NW, Calgary, T2N 4Z6 Canada; 3 Department of Mathematics and Statistics, Faculty of Science, University of Calgary, 612 Campus Place NW, Calgary, T2N 4N1 Canada; 4 Biomedical Engineering Graduate Program, University of Calgary, 2500 University Drive NW, Calgary, T2N 1N4 Canada; 5 Alberta Children’s Hospital Research Institute, University of Calgary, 3330 Hospital Drive NW, Calgary, T2N 4N1 Canada; 6 Alberta Diabetes Institute, University of Alberta, Li Ka Shing Centre for Health Research Innovation, Edmonton, T6G 2E1 Canada; 7 Centre for Bioengineering Research and Education, University of Calgary, 2500 University Drive NW, Calgary, T2N 1N4 Canada; 8 Arnie Charbonneau Cancer Institute, University of Calgary, 3280 Hospital Drive NW, Calgary, T2N 4Z6 Canada

**Keywords:** Design of experiments, DOE, Stem cell, Bioprocessing

## Abstract

*“To consult the statistician after an experiment is finished is often merely to ask him to conduct a post mortem examination. He can perhaps say what the experiment died of.” – R.A. Fisher*

While this idea is relevant across research scales, its importance becomes critical when dealing with the inherently large, complex and expensive process of preparing material for cell-based therapies (CBTs). Effective and economically viable CBTs will depend on the establishment of optimized protocols for the production of the necessary cell types. Our ability to do this will depend in turn on the capacity to efficiently search through a multi-dimensional problem space of possible protocols in a timely and cost-effective manner. In this review we discuss approaches to, and illustrate examples of the application of statistical design of experiments to stem cell bioprocess optimization.

## Background

Stem cells are capable of both replenishing their own numbers, and giving rise to one (unipotent stem cells) or more (multipotent, pluripotent or totipotent stem cells) other cell types. As such, bioprocesses that produce these cells cost-effectively, in quantity and with the desired properties, are foundational to efforts to bring tissue engineering and regenerative medicine to the clinic.

Once basic research has provided proof of concept for specific cell-based therapies (CBTs), applied research into the conversion of bench-scale protocols into optimized bioprocesses comes to the fore. Promising early clinical trials to treat retinal degenerative diseases with embryonic stem cell (ESC)-derived retinal pigmented epithelium have showed encouraging results [1, 2] and have in turn led to further trials that attempt to use CBTs to treat these diseases (reviewed in [3]). Insulin-secreting beta-like cells derived from from ESCs are also undergoing phase I/II clinical trials to evaluate their efficacy as a CBT for Type 1 diabetes (trial ID NCT02239354). There are, however, a number of challenges that must be overcome before CBTs can become generally available. Biological, technical and economical factors that need to be addressed have all been expertly reviewed elsewhere [4–7]. These factors ought to be kept in mind even at the earliest stages of stem cell research to facilitate translation towards technically and economically viable CBTs. Two critical but often-overlooked metrics for a given stem cell bioprocess are yield, the quantity of output cells of the desired type produced, and sensitivity, the robustness of the process in the face of minor variations in input variables.

Protocol yield – cell production per input cell, per mL of growth medium, per unit cost, etc – is not widely reported in the stem cell literature, but forms an essential step in the understanding of process efficiency. Where the term efficiency is encountered, it is often conflated with the purity of the output population. This is a critical metric in its own right, particularly when as few as 1 in 4000 undifferentiated pluripotent stem cell (PSCs) can lead to teratoma formation [8] for example, but should be distinguished from process efficiency. Monitoring and process refinement around yield can enable dramatic improvements, once this point is recognized [9]. When considering the magnitude of cells required for the replacement of cell-dense organs, estimated to be upwards of 10^9^ cells per patient per treatment [10], the importance of yield to process viability becomes clear. Given a doubling time of approximately one week during early human fetal development [11], a 90-day protocol beginning with one million input cells should theoretically generate in excess of 7^9^ progeny, assuming continuous replication in the absence of cell death. While this example demonstrates that the quantities of material required for CBT are attainable in principle, it must also focus attention on opportunities for improvement in processes that fall short of these numbers. To have impact beyond the laboratory, stem cell bioprocesses will require yield optimization across a broad array of input parameters.

**Fig. 1:**
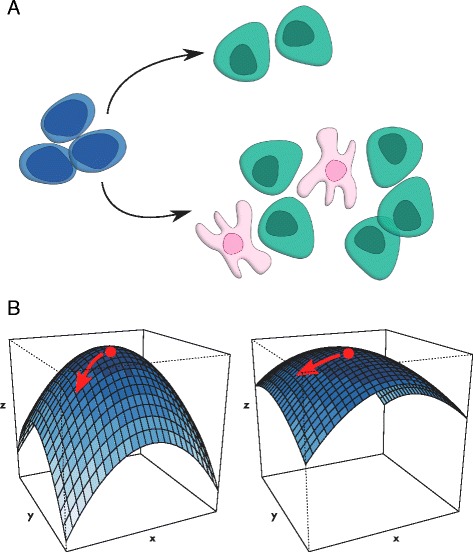
Yield and Sensitivity in Bioprocesses. Despite a high purity (top), it is important that the absolute yield (output cell per input cell; bottom) is also taken into account for a bioprocess to be commericially viable (**a**). Depending on the sensitivity of the system, the same change in one variable (here indicated by a shift along the x-axis) can result in very different responses (z-axis), a parameter that is important for reproducibility of a given process (**b**)

In turn, sensitivity directly impacts process reproducibility, currently a major concern in scientific publishing [12]. Cases of scientific fraud notwithstanding, it is likely that for the majority of processes that might be considered poorly reproducible, they exist in a highly sensitive region where small variations in one of potentially many process inputs (e.g. bioactive cytokine concentration, oxygen tension) can lead to drastic changes in output (Fig. 1). Where simple publication of an unreliable protocol can have negative reputational effects and lead to lost time and resources, attempts to translate such a protocol to the clinic can have far-reaching impacts on both patient health, and the financial viability of the organization responsible. Understanding to which inputs the process is most sensitive is essential for both good science, and the robust and reliable production of cells for therapeutic applications.

A review by Placzek et al. details many of the design principles required to translate stem cell bioprocessing into viable commercial products. Considerations toward process components such as cells and scaffolds, and process requirements including automation, characterization, harvesting and storage are detailed thoroughly [13]. The complexity of stem cell bioprocessing requires the examination of these multiple components that must be controlled to arrive at the correct state of the cell at the end of the process. Given this, it is important that careful thought be given to the design of experiments used to understand stem cell bioprocessing systems. Statisticians have been giving serious thought to such issues for many decades, developing a field of research known as design of experiments (DOE) or experimental design [14].

DOE methods cover a range of activities that relate to the logical choice of experiments with which to explore a system or test hypotheses about a system. In this review we highlight some important concepts of experimental design, and show how incorporating DOE techniques into stem cell bioprocessing can help answer fundamental questions about stem cell biology and facilitate the translation of basic and proof-of-concept research in stem cell bioprocessing.

## Design of experiments

### Background

In a basic research setting, experiments are commonly planned in an informal, ‘intuitive’ manner. Traditional experimentation in stem cell biology, as elsewhere, has typically been conducted using a one-factor-at-a-time (OFAT) approach. Under such an approach, attempts are made to hold every factor (variable) constant except for the target of investigation as this one factor is varied and the resulting output measured. This method can elucidate important biological ‘main effects’, but important effects from interactions between factors end up as part of the error term. Additionally, the complexity of stem cell bioprocessing requires the examination of numerous input variables that must be controlled to arrive at the correct state of the cell at the end of the process. While many investigations into optimized stem cell bioprocessing have used the OFAT method to substantially improve both purity and yield [9, 15–21], the involvement of multiple inputs (e.g., signaling pathways, oxygenation, duration of individual steps and the overall process, shear effects) means that understanding the interactions between factors will be necessary to optimize increasingly complex protocols.

**Fig. 2:**
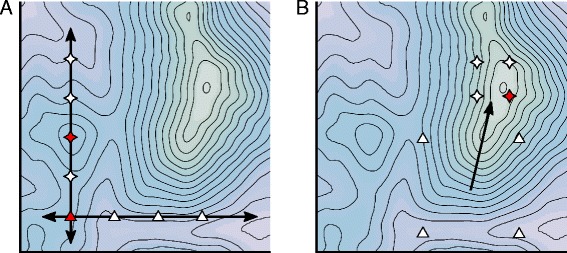
Two factor optimization and exploring the design space. Each axis represents a factor to be optimized for a given process whose output is shown as a contour plot, where each contour line represents a constant response (**a, b**). Determining the optimum using one-factor-at-a-time (OFAT) method first requires varying one factor (triangles) along the first axis to locate the maximum (red triangle). Once this is determined, a second set of experiments (stars) is performed by varying the second factor until its maximum is reached (red star). While the output has been increased, the true optimum in the space has not been reached (**a**). Conversely, starting with a widely spaced factorial experimental design allows for the evaluation of both variables in the first experiment (triangles) and statistical analysis can determine the path of steepest ascent (arrow). This is followed by a second set of experiments (stars) which can better locate the maximum output in the design space (red star) (**b**)

Consider the optimization of two variables in a stem cell bioprocess as shown graphically in Fig. 2. An OFAT approach would take us first in the direction of one axis, and then once optimized along this axis, perpendicular in the direction of the other. If we have luck on our side, and begin our exploration in a sensible place, we can arrive at the global maximum, thus finding settings of the two input variables tailored to optimizing our output variable. However, more likely, at the end of the experimental process we would find ourselves to be in fact at a local maximum or pseudo-optimum (as in Fig. 2 a). A better solution to finding the optimum could be achieved by considering a more thoughtful two factor experiment, or factorial design (Fig. 2 b). Such an approach, as well as leading to a better estimate of the optimum, also allows interactions between important variables in the culture to be estimated. A more rigorous process of determining where to place these experimental points and how to analyze the response is discussed below.

### Response surface methodology

In many situations experimental outputs can be noisy, and there may be many inputs of interest. In such cases, statistically-based experimental planning can result in much more informative data, in the sense that the selection of data points can be tuned to maximize information content relevant to the research questions of interest. The typical framework in which the DOE problem is set consists of *k* factors that are believed to have the potential to influence a given process output, *y*. Typically, each factor is assigned a small integer number of levels, *l* (e.g, {0,1} for *l*=2, or {-1, 0, 1} for *l*=3). The choice of experimental design then depends upon which among the many possible designs optimizes some criteria quantifying the amount of information that can be expected. This criterion is often based upon the precision or accuracy of the input variable estimates or predictions that can be made from the fitted model about the output variable.

We first consider the relationship between the output *y*, and each of our factors *x*_1_,*x*_2_,...,*x*_*k*_. In stem cell bioprocesses, the exact nature of this relationship is most often unknown. Instead, we generate an appropriate model of the system wherein we attempt to describe the output, or response, of the system based on potentially influential factors. This ‘response surface’ model is usually a first-order (linear) or second-order (quadratic) polynomial, and is generally based on continuous inputs such as temperature, serum concentration, levels of cytokines, and so on. Each variable is usually ‘coded’ so as to vary over the same range (e.g., {-1,0,1}) with mean zero and the same standard deviation [22]. The appropriate experimental design and matched analysis together consitute response surface methodology (RSM).

#### Sequential experimentation

**Fig. 3:**
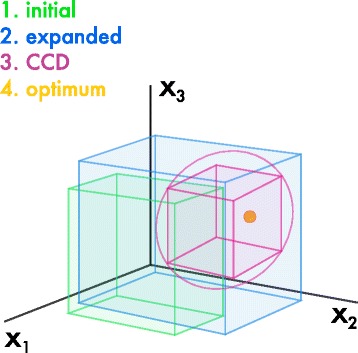
Sequential experimentation in RSM. A two-level factorial design can be used to initially assess the design space for three factors (*x*_1_, *x*_2_, *x*_3_; green). A fractional-factorial design would be more appropriate for processes with many more variables, see text for details. If model predictions suggest that the optimum may be outside of this range, an expanded factorial design can then be run (blue). Once we are confident that the optimum is located within our design space, a more complex CCD experiment can be run in a smaller area of the design space to provide a more accurate model of the process that includes non-linear responses (pink). Finally, the optimum can be located (orange)

One of the most important characteristics of RSM is the ability to design and analyze experiments sequentially. Initially, the experimenter will have ideas about which factors likely influence the response. An early stage screening experiment can verify the role of each factor and eliminate unimportant ones. This has the effect of reducing the number of factors for future experiments to limit the number of required experimental runs. Similarly, the fitted model is used to determine if the collected data lie near to an ideal response or at some distance from it. This allows for an investigation of the problem space and identification of where subsequent regions of experimentation should take place. At this stage, widely spread data points aid in developing an overview of the process space (Fig. 2 b). The final round of experimentation takes place around the true optimum and is designed to generate a model that more accurately represents the true function within a reduced problem space (Fig. 3).

#### Modeling

Each iteration of the experimentation serves to improve our model of the process. Beginning with a screening experiment, the important inputs can be determined and we thus have the building blocks for the model. Mathematical modeling of biological systems maximizes the information available from limited experimental data, and can help answer complex outstanding biological questions and understand nonintuitive behaviour [23–25]. As mentioned, it is important that the experimental data points are carefully collected. In order to take advantage of the statistical analyses implicit in RSM, experimental runs need to be conducted to produce a model that has strong predictive capabilities.

### Experimental designs

#### Factorial designs

**Fig. 4:**
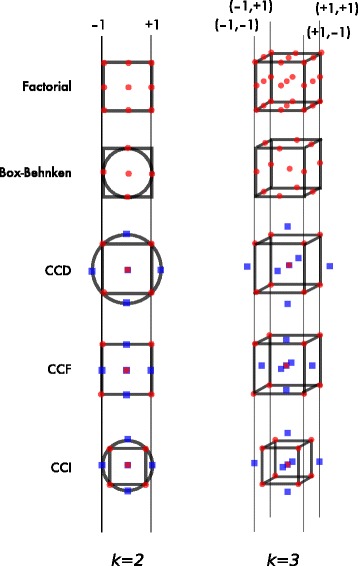
Examples of experimental designs with two and three factors. Factorial designs are constructed by testing every factor at each level, and can lead to large numbers of experimental runs as the number of factors increases. Box-Behnken designs reduce the number of experimental runs, but interactions between factors at ‘extreme’ levels are not included (i.e., the ‘corners’). Box-Wilson or Central composite designs (CCDs) combine factorial designs with ‘star points’ (shown in blue) to estimate second-order (quadratic effects). Modifications to the CCD result in face-centred (CCF) or inscribed (CCI) designs when the design space must be constrained

In a factorial design, each experimental run consists of a combination of levels for each factor. A full factorial design requires each combination of each factor at every level to be run, resulting in *l* ^*k*^ experimental runs (often 2^*k*^ or 3^*k*^). However, such designs can become very large in size. If we have two three-level factors, the full factorial design consists of nine experimental runs. As we increase the number of three-level factors, the full factorial requirement increases to 27, 81, 243, 729, 2187, etc. runs (Fig. 4).

A fractional factorial experiment makes use of a subset of these runs, *l* ^*k*−*p*^, where *p* is the size of the fraction of the full factorial. Fractional factorial designs can be used to investigate the most important aspects of the design space with considerably less effort and cost than would be required for a full factorial experiment. In general, we choose a fractional factorial design where some of the high order interactions are assumed to be negligible, but we can still estimate main effects and lower order interactions. Provided the same signaling pathway is not targeted by multiple variables, we would not commonly expect third-, fourth- or higher-order interactions between the variables to significantly affect biological changes [26]. Instead, by modeling first- and second-order interactions, we capture the most critical components of the bioprocess.

#### Central composite designs

Moving from full or fractional factorial designs we begin to encounter five-level experimental designs commonly refered to as Box-Wilson, or central composite designs (CCDs) [27]. These designs allow for the efficient estimation of second degree polynomial and quadratic responses [27]. Central composite designs attempt to balance the design, through the use of coded variables, to achieve rotability. By removing directional bias in the design, rotable designs predict output values with the same precision at all factor levels a constant distance from the centre of the design. These designs possess a high level of orthogonality, which means that each coefficient estimate is independent from one another [27]. Starting with a fractional factorial design, CCDs extend the range of each variable through so-called ‘star points’ that allow for the estimation of curvature. Therefore, CCDs are a five-level design, { −*α*,-1, 0, 1, *α*}. Two important classes of CCD with regards to stem cell bioprocessing are those designs that limit the experimental space to known regions rather than extending *α* (star points) potentially outside of realistic ranges (e.g. negative cytokine concentrations). These are known as central composite inscribed (CCI; whereas the original designs were circumscribed) and face-centred (CCF) designs. Examples of CCD, CCI and CCF designs for two and three factors are shown in Fig. 4. Importantly, in all types of CCDs, the uncertainty of the model predictions increases markedly as factor levels approach the upper and lower ends of the ranges investigated [28]. This highlights the advantage of sequential experimentation to re-centre the design and generate a more accurate model around the suspected optimum.

#### Advanced experimental designs

With continuing increases in computer power, more complex designs for nonstandard scenarios and models can also be produced. In the designs described above, the number of runs used is generally constrained by mathematical considerations. For example, in a five-factor, two-level factorial scenario, the full factorial design consists of 32 runs. It is trivial to construct half fraction factorial designs of 16 runs, or quarter fraction designs of eight runs. However, it is not easy to construct a design of say 15 runs using such methods. However, in so-called optimal design, an optimality criterion is selected, usually based upon the precision of the parameter estimates or model output. The computer is then used to carry out a search of possible designs for a set number of runs chosen by the user. This can be computationally intensive, but allows the user a much greater deal of flexibility in setting their design parameters. For example, any set number of runs can be chosen according to logistical constraints of the process or system being examined, and in situations where various factor level combinations are infeasible, irregular design spaces, which do not include such factor level combinations, can be constructed.

Further, when we wish to fit nonlinear/polynomial models (e.g., theoretically derived growth curves for biological processes) to our experimental data, an added complication to the design problem is that the optimal design will now depend upon the parameters of the underlying model. This poses a circular problem since we are wishing to construct a design to estimate the parameters of the underlying model, but we need to know the parameters of the underlying model in order to find the optimal design. A typical approach to such problems is to use Bayesian optimal design (e.g., [29]), in which a prior distribution has to be placed on the model parameters, expressing the user’s belief and uncertainty about the parameters before the data was observed. Such approaches can be carried out in a sequential manner so that at subsequent iterations of the design and analysis process, we can hone in on the salient regions of the design space and improve upon the quality of the fitted model.

## Design of experiments and stem cell bioprocessing

### Stem cell growth and expansion

Given the ability of DOE approaches to model complex behaviour, many aspects of stem cell bioprocessing would benefit from the application of these techniques. Although the adoption of DOE into stem cell bioprocessing has been limited, its use has started to expand in recent years. Of particular note are those investigations looking at stem cell production.

An early investigation into the 10-day in vitro expansion of haematopoeietic stem and progenitor cells (HSCs/HPCs) isolated from adult mouse bone marrow used a two-level full factorial design to screen the effects of cytokines, and the incubation temperature [30]. Following this initial screen, a more detailed analysis of interactive effects on the desired cell population was undertaken using response surface methodology [30]. This was used to develop an empirical model describing HSC repopulation, colony formation, and total cell expansions as a function of three cytokine concentrations. Each of the fractional factorial designs was composed of 16 experimental units plus four replicated points (center points), to obtain an independent estimate of the intrinsic variability (pure error) in the data [30]. Synergistic interactions between interleukin-11 and flt-3 ligand on total cell production was also detected, as was a negative third-order interaction between all three cytokines. These negative interactions reflect the fact that the combined effect on total cell and colony-forming cell production was less than the sum of their individual effects [30]. This study extended other single factor studies and identified important interactions in a complex multiple interacting cytokine culture system.

With the goal of defining the operating space for economic passaging of human ESCs, a three-level, three-factor (i.e., 3^3^) Box-Behnken experimental design was applied to evaluate the effects of seeding density, media volume and media exchange time [31]. Experimental data were subsequently used to model two-process responses: ESC expansion performance at the second passage and at harvest (24 h later) [31]. The authors found that lack-of-fit tests were not significant, indicating that additional variation in the residuals could not be removed with a better model [31]. Initially, three Box-Behnken RSM cell culture experiments, incorporating the chosen factors at software-specified design levels, were conducted over 36-, 48- and 60-h passage periods, although analysis of the models with a 48- and 60-h passage period did not provide outcomes that met critical optimization criteria [31]. Interestingly, they applied mathematical multiple-response optimization routine (desirability analysis) to visualize the region where both responses were simultaneously within optimization criteria [31]. While the authors of this paper acknowledged the use of T25 flasks during their ESC culture, they support the use of this method as a direct step-up to automated T-175 processes, as the cells were passaged using a single-cell method amenable to automation.

It is indeed of critical importance to be able to automate the process, as traditional planar culture is labour-intensive and will make CBTs unrealistically time consuming and expensive. Thomas et al. used an automated system combined with a full factorial design to optimize media concentrations for the expansion of human MSCs. Their use of a full factorial was necessitated by a need to avoid confounding interactions with main effects [32]. An alternative approach could have been an initial fractional factorial experiment, to identify those factors most important in the expansion of this cell population, before switching to a more refined, composite design that would permit investigation of both interactions and quadratic effects in the system. Nonetheless, this proved to be an interesting study that examined key components necessary in the expansion of MSCs including cell seeding density, serum percentage, media volume per flask, and culture time [32]. Interestingly, they found that seeding density and serum level had negative interactions, yet high levels of one or the other improved cell growth. The use of automation and robotic culture allowed for improved randomization of runs and removed many sources of variation from human processing of each flask.

While automated planar culture may prove sufficient for CBT development, particularly relating to monolayer tissues such as the retinal pigmented epithelium, the production of large numbers of stem cells has largely been left to stirred suspension bioreactors. Their capacity for empirical scale up, compared to other systems, and the ability to precisely regulate the culture environment in real time makes them ideal candidates for DOE applications. Because of variations in impeller design and the precise geometries of each bioreactor, little consistency is found between published protocols for the expansion of stem cells using bioreactor technologies. Hunt et al. undertook a full factorial design (3^2^) to investigate the effects of inoculation density and agitation rate on the production of human ESCs. It was found that the interaction of these two factors had a significant effect on growth rate, and to a lesser extent the maximum density [33]. Interestingly, higher inoculation densities negatively affected the fold increase [33]. While this study was limited in its scope, it revealed important interacting effects that may not have been uncovered using a typical OFAT approach. In both planar cultures and stirred suspension bioreactor systems, DOE can be applied early on to understand the process and this may subsequently advise for or against one particular system. When a particular production system is chosen, further application of DOE will allow for optimization of the bioprocess depending on the specific outputs desired.

#### Biomaterials

Most often, experimental design has been applied to biotechnologies that have considerable chemical and engineering components. For instance, Zhou et al. used several designs to optimize the degradation of gelatin-PEG composite hydrogels [34]. After first screening factors with a Plackett-Burman design, these same factors were used in a Box-Behnken central composite design to understand the interaction between them and generate response surfaces for systematic optimization [34]. While they did analyze the survival of MSCs seeded onto these hydrogels, only the degradation rate was used as an output parameter. With the model established, it would have been interesting to include viability of MSCs seeded as a response output to better understand the design space. Nih et al. also used a DOE approach to create a complex in vitro matrix environment with varying peptide motifs and growth factors [35]. Neural precursor cells derived from iPSCs were encapsulated in hydrogels and exposed to combinations of brain-derived neurotrophic factor (BDNF) and BMP-4 using in vitro neural cell survival as an output before the optimized gels were tested in vivo in an induced stroke mouse model [35]. As a brief data communication, there was little discussion of the effects of using DOE to generate a hydrogel, although heparin modification of the hydrogel interacted with the concentrations of growth factors, showing that low BDNF and low BMP-4 was beneficial when heparin was bound as opposed to high BDNF in non-heparin conditions [35].

A more thorough investigation of hydrogel formulation was demonstrated using modular self-assembling peptide ligands to generate synthetic extracellular matrices (ECMs) [36]. Jung et al. exploited the modularity of the system to undertake factorial experiments and RSM, and avoid the compositional drift that occurs when changing the concentrations of one molecule without affecting the concentration of others. They first began by testing each ligand alone to determine independent effects on endothelial growth. This was followed by a factorial design to identify interactions between ligands before using a CCI design to optimize their formulation [36]. At each stage of experimentation, the design space was shifted towards the perceived optimum. This study elegantly demonstrated a sequential experimentation strategy that was able to significantly improve cell growth on their optimized synthetic ECM upwards of 30% over their pre-optimized formula [36]. Interactions between nearly all ligands was found to be significant, with the strength of the effect of one ligand dependant on the concentration of another [36], lending more weight to the desirability of avoiding OFAT approaches to optimize biomaterial formulations.

#### Stem cell differentiation

Whereas most multifactorial studies look at stem cell expansion and survival, Chang and Zandstra, and Glaser et al. have showed that models of the differentiation process can also be fitted and optimized using DOE techniques.

Directing the differentiation of ESCs towards a definitive endodermal fate, two rounds of experiments using factors from the literature were conducted [37]. These were: glucose, insulin, basic fibroblast growth factor (bFGF), epidermal growth factor (EGF) and retinoic acid (RA), and the output of the system was measured in terms of the percentage of cytokeratin-8 and hepatocyte nuclear factor-3 *β* double-positive cells obtained after thirteen days [37]. After identifying the most important factors in a two-level, five-factor factorial experiment (2^5^), the authors conducted a refined three-level, two-factor factorial experiment (2^3^) to identify synergistic and quadratic effects of RA and EGF, holding the other factors fixed. As this study’s purpose was to identify a quantitative screening technology, differentiation protocols were not further optimized [37]. This study, did nevertheless reveal interesting interactions between these factors that had varying effects on each of the different outputs, namely total cells, total endoderm cells and the percentage of endoderm cells with RA and the interaction between glucose and RA negatively impacting all three processes [37].

Using their previously published chemically defined protocol for generating endothelial cells from ESCs, Glaser et al. included a number of factors in their optimization: time, cell seeding density, matrix substrates and cytokines [25]. They used a two stage differentiation protocol to direct endothelial cell fate, first generating mesodermal vascular progenitor cells (VPCs) before final endothelial cell (EC) differentiation, each run as a full factorial experiment and assessed by the expression of Flk-1/KDR ^+^ VPCs (mouse and human marker, respectively) and VE-cadherin ^+^ ECs [25]. Fibronectin and seeding at 10,000 cells/cm^2^ was shown to generate the greatest number of VPCs in both human and mouse ESCs. Interestingly, this group also assessed the importance of time in differentiating pluripotent cells and found that induction of Flk-1/KDR occurred within a short time window before receding [25]. Lower seeding of mouse VPCs (5000-10,000 cells/cm^2^) on fibronectin with high concentrations of bFGF (50 ng/ml) resulted in up to 95% ECs, whereas human VPCs generated ECs at a rate of 57% when seeded on gelatin with considerably lower bFGF (10 ng/ml). While vascular endothelial growth factor was shown to be statistically unimportant at all stages of EC differentiation, significant interaction effects between seeding density or bFGF concentrations and culture matrix were observed [25]. Follow-up experiments using the generated model-based predictions were not tested directly, but rather lined up with the closest experimental run to determine the optimal conditions for generation of ECs. However, this investigation did provide a considerably larger set of variables to be optimized for directing stem cell differentiation.

## Conclusions

A major strength of DOE methodology – and RSM in particular – lies in the ability to build on carefully designed experiments in a sequential manner. In stem cell bioprocessing, these sequential experiments can lead to the construction of an empirical model that can elucidate fundamental processes related to cell biology as well as provide a foundation from which future experiments and translational research can take place. Generating mathematical models of the process with carefully planned experiments maximizes information about the system.

As detailed above, models of a given system are of great value to understanding the nature of stem cell biology, and have revealed important insights that can be missed with traditional OFAT methods of experimentation that are less able to study interactive effects between various growth parameters [30]. When applied to the complex systems of stem cell biology, DOE provides an important tool to unravel important interactions. Equally important in science more generally is the ability for experiments to be replicated. Understanding the design space, the importance of specific parameters on the outcome and how robust the entire process is, provides guidance on the reproducibility of the system. Adoption of DOE techniques to help model the system inherently provides a means to test sensitivity and an understanding of how reproducible a given result is likely to be. This in turn will facilitate the translation of fundamental research into viable CBTs. Industrial processes, including the production of cells as therapies, will require robust operating parameters to deal with inevitable variation in batches of input cells, for example. Understanding the system’s sensitivity, or pressure points, is necessary to engineer safeguards preventing failure during production runs.

Continued research into stem cell bioprocesses will greatly benefit from the application of DOE methods. There are, however, still challenges with its implementation in a high throughput manner, particularly with regard to identifying suitable cell outputs, such as marker expression or functional assays. Traditional assessment of cell behaviour by immunostaining, for example, are generally considered unsuitable for large scale screens. However, recent advances in high-content screening have begun to make this a viable analytical method [37, 38]. Development of biosensors and ’omics technologies and their integration into stem cell bioprocessing pipelines will help to overcome these challenges. Coupled with real time monitoring of bioreactor cultures and automation of routine cell culturing procedures, it should soon be possible to screen large numbers of inputs to generate robust stem cell bioprocesses built on DOE methodology. The use of DOE in other bioprocessing fields such as the production of enzymes and other proteins has continued to grow [39]. As CBTs move towards the clinic, the incorporation of DOE into stem cell bioprocessing will provide a stable foundation upon which therapeutic applications may confidently by constructed.

## Abbreviations

bFGF: Basic fibroblast growth factor
CBT: Cell based therapy
CCD: Central composite design
CCF: Central composite face-centred
CCI: Central composite inscribed
DOE: Design of experiments
EC: Endothelial cell
ECM: Extracellular matrix
EGF: Epidermal growth factor
ESC: Embryonic stem cell
HPC: Haematopoeietic progenitor cell
HSC: Haematopoeietic stem cell
OFAT: One-factor-at-a-time
PSC: Pluripotent stem cell
RSM: Response surface methodology
RA: Retinoic acid
VPC: Vascular progenitor cell
